# How does COVID-19 pandemic affect entrepreneur anxiety? The role of threat perception and performance pressure

**DOI:** 10.3389/fpsyg.2022.1044011

**Published:** 2022-10-19

**Authors:** Yunjian Li, Hongchuan Chen, Chunzhen Liu, Hong Liu

**Affiliations:** School of Management, Guangzhou University, Guangzhou, China

**Keywords:** COVID-19 pandemic, entrepreneur anxiety, entrepreneur threat perception, entrepreneur performance pressure, entrepreneurial firms

## Abstract

The entrepreneurial firms may be more vulnerable to the COVID-19 pandemic, and the entrepreneurs of entrepreneurial firms are also threatened by the revenues decline and business failure, which vehemently affect their well-being. The mental health of the entrepreneur decides whether the entrepreneurial firms can make the right decision, which is related to the healthy development of the entrepreneurial firms. Based on the event system theory and the cognitive appraisal theory, this paper aims to explore the effect of COVID-19 pandemic on the entrepreneur anxiety, and the threat perception and performance pressure are introduced to investigate the mediating mechanism and boundary of this effect. Using the simple random sampling to obtain questionnaire survey data, 168 entrepreneurs of entrepreneurial firms have participated in the empirical study, and the research results are as follows. First, the COVID-19 pandemic significantly positively affects entrepreneur anxiety. Second, the entrepreneur threat perception plays a mediating role between the COVID-19 pandemic and the entrepreneur anxiety, which means the COVID-19 pandemic can enhance the external threat perception of entrepreneurs, and then affect the entrepreneur anxiety. Third, the positive effect of the COVID-19 pandemic on the entrepreneur anxiety is strengthened by the entrepreneur performance pressure, while the positive effect of entrepreneur threat perception on entrepreneur anxiety is weakened by the entrepreneur performance pressure. The above findings are helpful to explore the mechanism of the COVID-19 pandemic and other critical crisis events on entrepreneurs’ mental health from the new perspective of cognitive appraisal theory and event system theory, filling the research gaps between the COVID-19 pandemic and entrepreneur anxiety. Besides, this study broadens the applied range of the cognitive appraisal theory and the event system theory in the fields of crisis situations and entrepreneur research, and enriches the research outputs. Furthermore, this study will help provide a new theoretical analysis insight for the related research on how the COVID-19 pandemic affects entrepreneurs’ psychology, and further deepen researchers to understand the mechanism of entrepreneur anxiety under the COVID-19 pandemic, providing theoretical inspirations for reducing entrepreneur anxiety. What’s more, this study finds that individual pressure can affect their cognitive appraisal, which means that future research should take the pressure influential mechanism into consideration in the process of exploring “external stimulus--cognitive appraisal--emotional response,” further expanding the theoretical model of cognitive appraisal proposed from the perspective of pressure.

## Introduction

Entrepreneurial Firms *(EFs)* have become a key driver of economic growth and job creation (OECD, 2020). The outbreak of the COVID-19 pandemic has exerted influence over economic development (Mckibbin and Fernando, 2021), threatening the survival and development of *EFs* (Hoang et al., 2022), and those entrepreneurs who play an important role in business development. Under the background of the currently poor entrepreneurial environment (Ramadani et al., 2022), in addition to the health risks of the COVID-19 pandemic, entrepreneurs are also threatened by economic shocks, the decline in business revenues and bankruptcies incurred by industry downturns, which affect their well-being (Xu and Jia, 2022). Under the influence of the COVID-19 pandemic, the entrepreneurs of *EFs* often face more severe financial pressure and uncertainty (Backman et al., 2021), which is not conducive to entrepreneurs to make the right decision for the sake of the long-term development of *EFs*. Therefore, it is of great significance to understand the influential mechanism of the COVID-19 pandemic on entrepreneurs’ mental health and to provide effective intervention measures.

According to the existing literature, some studies have probed into the relationship between the COVID-19 pandemic and entrepreneurial psychology (Backman et al., 2021; Mustafa et al., 2021; Torres et al., 2021; Chhatwani et al., 2022; Sornsenee et al., 2022). For instance, a survey of 816 SMEs’ entrepreneurs by Lathabhavan (2022a) finds that economic difficulties and financial threats caused by the pandemic are main causes of anxiety, pressure and depression among entrepreneurs. Xu et al. (2021) take small and micro entrepreneurs as research objects, discovering that economic pressure, uncertainty, operation-related pressure and social pressure are main sources of pressure. Xu and Jia (2022) find that the past performance of a start-up will reinforce the negative impact of the COVID-19 pandemic on the mental health of entrepreneurs through a tracking survey of 303 entrepreneurs in start-ups. The above studies mainly analyze the factors affecting the mental health of entrepreneurs under the background of the COVID-19 pandemic. Despite of some progress achieved, the following research gaps still remain.

First, from the perspective of research object, the researches regarding the impact of the COVID-19 pandemic on entrepreneurs’ psychology focus more on SMEs (Chhatwani et al., 2022; Sornsenee et al., 2022; Lathabhavan, 2022a), and less on entrepreneurs of *EFs*. Moreover, *EFs* play a crucial role in job creation, radical innovation, and long-term growth (OECD, 2020), yet since at a fragile stage in their life cycle, they may be more vulnerable to the COVID-19 pandemic (Benedetti-Fasil et al., 2022), and its founders are more likely to experience psychological problems. Therefore, this paper takes the entrepreneur of *EFs* as the research object to explore the COVID-19 pandemic’s influential mechanism on the entrepreneur anxiety (*EA*), hoping it can provide the theoretical enlightenment for alleviating the entrepreneur psychological anxiety, and promoting the healthy development of *EFs* and economic recovery.

Second, from the perspective of research content, previous studies on entrepreneurs and *EFs* mostly emphasize on exploring their coping strategies under the influence of COVID-19 pandemic (Kuckertz et al., 2020; Jhajharia and Sharma, 2021; Dragan et al., 2022; Mota et al., 2022), but neglect to explore the influence of COVID-19 pandemic on entrepreneur’s emotion. Entrepreneurs as pivotal decision makers for their businesses, their mental health and well-being play a critical role in their decisions, motivations, and actions (Stephan, 2018). In view of this, this paper bends its attention on entrepreneurs’ emotions to explore the influential mechanism of the COVID-19 pandemic on it. Specifically, the psychological effects of the COVID-19 pandemic on individuals contain a wide range of worries, fears, and anxieties (Ahorsu et al., 2020; Wang et al., 2021; Moral et al., 2022; Lathabhavan, 2022b). Among them, anxiety is the most common negative mood with the COVID-19 pandemic. Anxiety is an emotional state of tension, restlessness, and worry caused by a threatening environment (Spielberger et al., 1976), and it can trigger and induce the corresponding behavior (Arnold, 1960), which in turn influences entrepreneurial decision-making and behavior (Stephan, 2018). Respecting this, this paper focuses on *EA*, and further discusses the influencing mechanism of the COVID-19 pandemic event’s strength on *EA*, thereby providing a theoretical basis for solving the psychological problems of entrepreneurs and promoting the healthy development of *EFs*.

Third, from the perspective of influential mechanism, existing researches have ignored the mutual impact of internal performance decline pressure and external environmental threats on *EA*. Performance is critical to a company’s long-term development, yet owing that *EFs* are still very young, they often have little revenue, operating deficits, and low chances of survival (Damodaran, 2009), tending to face more uncertainty. The COVID-19 pandemic has a tremendous, irregular, and sudden external impact on *EFs* and their operations (Liu et al., 2021), which leads to a drastic decline in sales, profits, and market share, finally resulting in a performance decline (Li et al., 2022). Different *EFs* are somewhat differently affected by the COVID-19 pandemic, so the expected firm performance decline of their entrepreneurs will be different, and the COVID-19 pandemic on their performance pressure (*PP*) will be different. Entrepreneurs perceive the performance pressure brought by the decline of enterprise performance, which is referred to as “entrepreneur performance pressure (*EPP*).” There may be differences in threat perception (*TP*) under different levels of *PP,* which probably have dissimilar effects on the entrepreneur’s emotion. Based on this, this paper introduces *EPP*, as a moderator to further explore the boundary condition of the influence of COVID-19 pandemic event’s strength on *EA* through entrepreneur threat perception (*ETP*).

In order to fill the research gaps mentioned above, this paper takes the COVID-19 pandemic event’s strength as the antecedent variable affecting the *EA*. On this basis, according to the cognitive appraisal theory proposed by Skinner and Brewer (2002), *TP* is regarded as the result of people’s cognition to external stimuli, and the antecedent of negative emotions such as anxiety as well. In this paper, *ETP* is introduced as a mediator variable of the COVID-19 pandemic event’s strength and *EPP* as a moderator variable to explore the influential mechanism of the COVID-19 pandemic on *EA*. Compared with the existing research, the potential research contributions of this paper are as follows.

First and foremost, it is different from the researches based on the conservation of resource theory (Torres et al., 2021; Xu and Jia, 2022; Lathabhavan, 2022a), stressor-detachment model (Backman et al., 2021), stress event theory and job demand-resource model (Torres et al., 2021), to explore the impact of COVID-19 pandemic on entrepreneurial psychology. In this paper, the cognitive appraisal theory and the event system theory are introduced into the existing research, and the COVID-19 pandemic is measured from three dimensions including novelty, disruption and criticality. By virtue of measuring entrepreneurs’ perceptions about the novelty, disruption and criticality of the COVID-19 pandemic from the perspective of events’ attributes, this paper examines the effect of the COVID-19 pandemic event’s strength on *EA* and its mechanism. Meanwhile, it offers new insight to reveal the impact of the COVID-19 pandemic on entrepreneurs’ psychology, and a new theoretical perspective to understand and solve the mental health problems of entrepreneurs.

Secondly, this paper expands the research objects of the cognitive appraisal theory and the event system theory. Current event systems theory is mostly used to analyze the impact of the COVID-19 pandemic on employees (Lin et al., 2021; Yin and Ni, 2021), while cognitive appraisal theory is used to study individual emotions or behaviors, attaching more importance to consumers (Watson and Spence, 2007), tourists (Hosany, 2012), and employees (Shagirbasha and Sivakumaran, 2021), yet less on entrepreneurs. Taking the entrepreneurs of *EFs* as the research object, this paper explores the influential mechanism of the COVID-19 pandemic on entrepreneurs’ emotion, concerning about the psychological state of entrepreneurs under the COVID-19 pandemic, which expands research objects and the scope of these two theories. It is also the first time to integrate the cognitive appraisal theory and event system theory together, which enhances the explanatory potency of the two theories in the study of events’ influence on individuals. Besides, it also broadens the scope of application of these two theories in the fields of crisis situations and entrepreneur research, and enriches the research outputs of it in the fields of the COVID-19 pandemic and entrepreneur research.

Thirdly, this paper introduces *ETP* as the mediator variable of the COVID-19 pandemic affecting *EA*, and *EPP* as the moderator variable. Furthermore, the boundary condition of the COVID-19 pandemic event’s strength affecting *EA* through *ETP* gets explored and clarified. Most previous studies have regarded *TP* as an antecedent variable of anxiety mood (Cypryanska and Nezlek, 2020; Paredes et al., 2021), and performance as a result variable (Mota et al., 2022) or a moderator variable (Xu and Jia, 2022). Based on the cognitive appraisal theory and the impact of the COVID-19 pandemic, this paper considers the COVID-19 pandemic as an independent variable of anxiety, and takes the mediating effect of *ETP* into account. In addition, considering that entrepreneurs have different threat perception and anxiety under different performance pressures, this paper takes *EPP* as a moderator variable. By introducing *ETP* and *EPP* as mediator and moderator variables, and incorporating pressure and *TP* into the same research model, extends the previous model that only considers the moderating role of past performance, proposed by Xu and Jia (2022), and the model proposed by Cypryanska and Nezlek (2020), which only considers the mediating role of *TP*. The results of this study will help provide a new theoretical analysis insight for the related research on how the COVID-19 pandemic affects entrepreneurs’ psychology, and further deepen researchers to understand the mechanism of *EA* under the COVID-19 pandemic, providing inspirations for reducing *EA.*

The structure of this paper is arranged as follows. The first section is an introduction, the second section is the theoretical analysis and research hypothesis, the third section is the research design, the fourth section is the empirical results, the fifth section is the discussion, and the sixth section is the conclusions.

## Theoretical analysis and research hypothesis

### Event system theory and cognitive appraisal theory

The impact of the COVID-19 pandemic is complicated. Whereas event system theory can incorporate context into theorization and provide profound methods to quantify the influence of events (Johns, 2017), it can facilitate to understand the complex impact of the COVID-19 pandemic. Event system theory highlights the impact of events on organizations and individuals in the light of the spatial, temporal and strength attributes (Morgeson et al., 2015). As for the COVID-19 public health emergency, the time, space and strength of the outbreak will exert distinct influence over organizations and individuals. Event’s strength can be measured in terms of its novelty, disruption, and criticality (Morgeson et al., 2015). Novelty reflects the extent to which an event differs from current and past behavior, characteristics, and events, representing a new or unexpected phenomenon (Morgeson, 2005). Disruption reflects the extent to which an event changes organizations and individuals (Morgeson, 2005). Event’s criticality reflects an event’s importance, necessity and priority for organizations and individuals (Morgeson and DeRue, 2006). Specifically, the novelty of the COVID-19 pandemic measures the unexpected and unusual degree for entrepreneurs. In terms of disruption, the COVID-19 pandemic has a significant impact on the catering industry (Byrd et al., 2021), the tourism industry (Jang et al., 2021; Kim et al., 2021), and people’s physical and mental health (Shah et al., 2022), and so on, which has changed production activities and brought changes in the external environment, representing the changing and influencing extent of the pandemic on entrepreneurs. As to criticality, the criticality of COVID-19 pandemic event measures the extent to which the COVID-19 pandemic is “important, necessary or prior” for entrepreneurs. This paper measures it from the strength of novelty, disruption and criticality, to explore the impact of the COVID-19 pandemic on the emotions of entrepreneurs.

The cognitive appraisal theory holds that emotions arise from an individual’s evaluation and cognitive process of a stimulus event (Smith and Ellsworth, 1985; Frijda, 1987; Roseman et al., 1990; Tomaka et al., 1997; Oatley and Johnson-Laird, 2014). Specifically, once a stimulus event is perceived, the individual will automatically make an evaluation of “whether it is good or bad for me,” which in turn generates an emotion associated with the stimulus event (Frijda et al., 1989; Lazarus, 1991). Therefore, the process of cognitive appraisal is the decisive factor of emotion production. Arnold (1960) contends that emotion is a tendency to access to something good or fond, or to stay away from something bad or sick, and that any evaluation of things or events is tinged with emotion or mood. After that, researchers have expanded and supplemented the cognitive appraisal theory by combining different scenarios. For example, Lazarus (1991) further extends Arnold (1960)’s emotional evaluation to a kind of evaluation including information screening, motivation, physiological changes and reactions, and other components, and re-appraisal process. Roseman (1996) extends the dimensions of the emotion evaluation model by estimating the situation state, motivation state, control potential, problem source, etc. Skinner and Brewer (2002) proposes a theoretical model of cognitive appraisal, arguing that emotions stems mainly from the appraisal and cognitive processes of threats and challenges, which means challenge appraisal is related to positive emotions while threat appraisal is involved with negative emotions. *TP* is not only the result of people’s cognition to external stimuli, but also the antecedent of negative emotions such as anxiety. Skinner and Brewer (2002) puts forward the cognitive appraisal theory model, which is a theoretical basis for our study. As to entrepreneurs, the COVID-19 pandemic is a kind of negative external stimulus event. The uncertainty and economic shock caused by the COVID-19 pandemic will bring about entrepreneurs pressure like external threats and the performance decline, which would lead to anxiety and other negative emotions to them. Based on the model proposed by Skinner and Brewer (2002), this paper takes *EFs* as the research object, the external stimuli of the COVID-19 epidemic as the independent variable, and *EA* as the dependent variable, *ETP* as mediating variable, *EPP* as moderator variable to explore the influential mechanism of the COVID-19 pandemic on *EA*.

### The COVID-19 pandemic event’s strength and *EA*

*EA* is rooted in the cyclical pursuit of entrepreneurial goals by individuals, mainly lying in persistent worry, suspicion and uneasiness about uncertain outcomes (Thompson et al., 2020). Besides, it derives mostly from a negative perception of environmental stimuli that threatens survival (Cacciotti and Hayton, 2015). In the context of the COVID-19 pandemic, the economic downturn and fluctuations in the external environment triggers threats to entrepreneurs (Torres et al., 2021). Apart from it, the uncertainty of the COVID-19 pandemic makes it easier for entrepreneurs to worry and fret about the future. Due to the initial insufficient funding and resources, those *EFs* who bear more economic pressure are more prone to being anxious.

The COVID-19 pandemic has issued in adverse and unforeseen problems for entrepreneurs (Meurer et al., 2022). Based on the event system theory, the impact of COVID-19 pandemic on entrepreneurs can be measured from three aspects: novelty, disruption and criticality. The novelty of the COVID-19 pandemic reflects the sudden and unexpected nature of the outbreak, and entrepreneurs do not have a well-established procedure and process to guide their actions. Under the circumstances, it is indispensable for them to explore and exploit new opportunities, or learn new skills, yet which will be difficult for them (Nummela et al., 2020), thus aggravating *EA*. The disruption reflects the changing degree in the routine entrepreneurial activities, chiefly involving the economic shock of the epidemic and the uncertainty of the external environment. Since the outbreak of the COVID-19 epidemic, the demand and supply chain of many enterprises have been affected (Bartik et al., 2020), and the ways of working and working environment have also changed (Costa et al., 2022). Online office at home is more widespread (Cuerdo-Vilches et al., 2021), which will limit the operation of a range of business, and even incur the closure of business (Torres et al., 2021), posing an economic threat. What is worse, economic threats can cause greater individual anxiety (Mamun et al., 2020). For *EFs*, working online may lead to higher financial costs, financial hardship and anxiety due to limited resources and funds. In addition, as the COVID-19 is highly contagious (Satici et al., 2021), which will further increase entrepreneur pressure and *EA*. The criticality of the COVID-19 pandemic reflects the extent to which it affects the long-term development of entrepreneurs. As for entrepreneurs, the COVID-19 pandemic not only disrupts their normal daily lives, but also endangers their situation and working modes (Nummela et al., 2020). Since the outbreak of the COVID-19 pandemic, it is predictable that the economic losses caused by the COVID-19 pandemic will be severe, ubiquitous and long-lasting (Torres et al., 2021). It will continue to affect the production and life in the future. The uncertainty of its duration will also incur pressure and uncertainty to entrepreneurs, which will render them to feel anxious. To sum up, the strength of the COVID-19 pandemic will increase *EA*, and then the following hypothesis is put forwarded.

*Hypothesis 1:* the COVID-19 pandemic event’s strength will positively affect *EA*.

### The mediating role of *ETP*

*ETP* is the entrepreneur’s evaluation and cognition in terms of external threats. Threat evaluation reflects an individual’s judgment that an external threat endangers their well-being, as well as an individual’s confidence degree and ability to cope with threats. The COVID-19 pandemic, as a public health emergency, has an impact on entrepreneurs’ subjective perceptions (Hernandez-Sanchez et al., 2020). First and foremost, due to the sudden and unexpected nature of the COVID-19 pandemic, entrepreneurs do not have a well-established procedure and process to deal with the sudden changes, and their normal production activities and lives are seriously affected, rendering them aware of threats. The infectiousness and severity of the COVID-19 itself may also increase risk perception among entrepreneurs. In addition, due to the high infectiousness and severity of the COVID-19, epidemic prevention measures need to be taken. When entrepreneurs think that these measures entails to be necessarily and quickly implemented, *TP* may be exacerbated (Tomaka et al., 1997). The COVID-19 pandemic also causes entrepreneurs unable to start businesses, resulting in lower sales and low profits (Li et al., 2022), which causes economic stress and *TP*. The criticality of the COVID-19 pandemic is a reflection of the extent to which it affects the long-term development of entrepreneurs and it threatens the achievement of their goals. The long-term impact of the COVID-19 pandemic crisis will remain unknown in the future (Korsgaard et al., 2020), so entrepreneurs do not know when the epidemic will end and how long it will last. These uncertainties will put pressure on entrepreneurs (Xu et al., 2021), which increases *ETP*.

The cognitive appraisal theory suggests that *TP* increases anxiety (Skinner and Brewer, 2002). Individual emotions are also closely related to their cognitive appraisal of the environment (Smith and Ellsworth, 1985). Anxiety occurs when an entrepreneur perceives a potential change and assesses that it threatens the achievement of personal goals, standards, or values (Thompson et al., 2020). The COVID-19 pandemic, as a negative external stimulus, will threaten the achievement of entrepreneurs’ personal goals, and then make them anxious. It is also widely accepted in existing studies that *TP* of the COVID-19 pandemic will induce psychological anxiety (Cypryanska and Nezlek, 2020; Paredes et al., 2021; Zhang et al., 2022), which in turn generates *EA*. Based on this, this paper argues that the COVID-19 pandemic will make entrepreneurs perceive external threats, and the greater the degree of external threats perceived by them, the more likely they are to become anxious. Afterwards, the paper proposes the following hypothesis.

*Hypothesis 2:* *ETP* plays a significant mediating role between the COVID-19 pandemic event’s strength and *EA*.

### The moderating role of *EPP*

The outbreak of the COVID-19 have led to supply chain disruption, raw material shortage, lower output and lower demand in many enterprises (Bartik et al., 2020; Sarker et al., 2022). It makes the enterprises operation threatened and even face the risk of temporary closure (Torres et al., 2021), bringing much uncertainty to enterprises’ business continuity (Zutshi et al., 2021). In this context, a large number of *EFs* have experienced varying degrees performance decline (Turner and Akinremi, 2020), which also puts entrepreneurs into varying degrees of *PP*. First of all, after the outbreak of the COVID-19 pandemic, for most enterprises, no matter how much resources they have invested before, the expected income they can get will fall to a lower level, bringing about greater losses for better-performing companies and a worse mood for entrepreneurs (Xu and Jia, 2022). Compared with the same period of previous years, the greater the gap between the expected performance year on year is, the greater the potential loss that entrepreneurs will face. The pressure increases as the performance declines. Anxiety occurs when entrepreneurs perceive and assess that the COVID-19 pandemic threatens the achievement of personal goals, standards, or values (Thompson et al., 2020). Compared with those entrepreneurs with low *PP*, the COVID-19 pandemic makes those entrepreneurs with high *PP* more negative mood, bearing greater losses. Therefore, after the COVID-19 pandemic, the latter are more likely to have high anxiety. In addition, the larger the expected performance decline is, the greater *EPP* will be. Whereas in high-stress status, anxiety is easier to be increased (Mesagno and Mullane-Grant, 2010; Ellis and Ward, 2022). Therefore, those entrepreneurs with high *PP* than low *PP* are more vulnerable to the negative impact of COVID-19 pandemic and have more anxiety accordingly.

The impact of the COVID-19 pandemic on *ETP* may be different with distinct *EPP.* From the perspective of resource, enterprise resources remain a necessary condition for enterprise operation and an important source for enterprises to obtain competitive advantage (Vartanova et al., 2021), which shows a significant positive effect on enterprise performance (Beleska-Spasova et al., 2012). If entrepreneurs’ expected performance this year is less than that of the same period in previous years, then they will deem that the enterprise resources are relatively insufficient. Those entrepreneurs with high *PP* confront a more serious deficiency of resources, threatening the enterprises’ operation, or even their survival, and then strengthening *ETP*. After the outbreak of the COVID-19 pandemic, it is more difficult for companies to obtain credit (Zhang and Fang, 2022), which exacerbates the problem of resource scarcity in enterprises (Garcia-Vidal et al., 2020). As for those entrepreneurs with high *PP*, it definitely makes things worse. In other words, the COVID-19 pandemic makes the lack of resources well-marked for entrepreneurs with high *PP*, which in turn will enhance their *TP.* From the perspective of the causes of *PP*, the individual’s evaluation on it depends on its cause, such as heavy workload, arduous task requirements, etc., rendering individuals to regard *PP* as a threat (Mitchell et al., 2019). As for the entrepreneur with high *PP*, the severe performance decline means that there remain serious problems in the enterprises’ operation, which makes them evaluate *PP* as a threat and enhance their *TP*. The COVID-19 pandemic has exacerbated the firm performance decline (Shen et al., 2020), further increasing *EPP*, so that entrepreneurs with high *PP* are more inclined to evaluate *PP* as a threat, and to enhance their *TP* brought by the COVID-19 pandemic.

*PP* will also affect the energy and mentality of entrepreneurs to deal with emergencies, and their ability to respond varies under different *PP* levels. Existing researches show that entrepreneurs’ capability and performance are positively and significantly correlated (Aujirapongpan et al., 2020; Qu et al., 2022), as those entrepreneurs with better opportunity recognition (Alim et al., 2022) and entrepreneurial ability (Xia et al., 2020) tend to have higher performance. The level of enterprise performance can reflect the capability of entrepreneurs to some extent. *EFs* with high performance in the past represent that they have stronger capability. Entrepreneurs with high *PP* expect performance to decline, indicating their sense of crisis. When the outbreak occurs, crisis-conscious entrepreneurs can more quickly perceive the threat of economic shocks and external uncertainties, and then analyze the current situation so as to quickly take measures to deal with the negative impact of emergencies. For entrepreneurs with high *PP*, in the face of the external threats posed by the COVID-19 pandemic, they are more likely to see them as a challenge based on perceived threats, so as to respond faster and improve their resilience. According to the cognitive appraisal theory (Skinner and Brewer, 2002), when individuals treat external threats as a challenge, they are more likely to generate a positive emotion, which can ease *EA* to some extent. To sum up, entrepreneurs with high *PP* will be able to rapidly take measures to tackle the crisis under the COVID-19 pandemic, thus soothing the anxiety caused by *TP*.

Based on the above analysis, this paper proposes the following hypotheses.

*Hypothesis 3a:* *EPP* positively moderates the relationship between the COVID-19 pandemic event’s strength and *EA*.

*Hypothesis 3b:* *EPP* positively moderates the relationship between the COVID-19 pandemic event’s strength and *ETP*.

*Hypothesis 3c:* *EPP* negatively moderates the relationship between *ETP* and *EA*.

**Figure 1:**
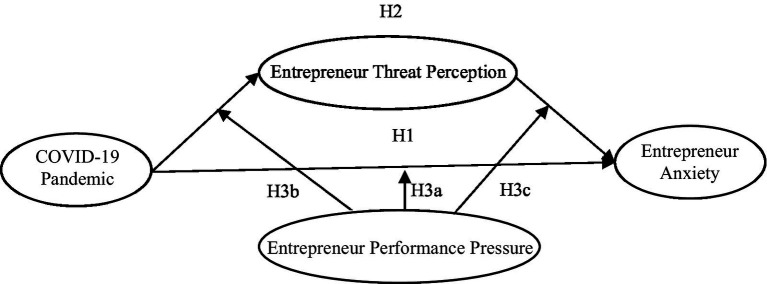
Research model.

Based on the above hypotheses, the research model is shown in Figure 1.

## Research design

### Sample

In view of the fact that it is difficult to measure the entrepreneurs’ psychological activities and anxiety with objective statistical data, this study uses a subjective questionnaire method to obtain analytical data. The survey is from June 2020 to September 2020 and the objects are those entrepreneurs of *EFs* from the science and technology parks in Guangdong Province, China. Because the simple random sampling works well in finding a generalized result that can be applied to the entire population (Rahman et al., 2002), this paper also chooses it to obtain the questionnaire survey data. The operation companies of the science and technology parks are entrusted to randomly distribute questionnaires to entrepreneurs of *EFs* in the parks. The survey has collected 206 questionnaires, excluding the enterprises that have been established for more than 8 years, there are 168 analysis samples of *EF*s. The sample enterprises mainly derive from the industries of information transmission, software and information technology services, scientific research and technology services. All of them are *EFs* within 8 years old with less than 300 employees and business income less than RMB 20 million, which shows that most of the sample enterprises are SMEs. And most of them have no overseas business, which fits the characteristics of *EFs* and has a good representative. The basic information of the sample enterprises is shown in Table 1.

**Table 1:** Characteristics of sample enterprises.

**Variables**	**Categories**	**Frequency**	**Percentage (%)**
Number of employees	Less than 20 employees	124	73.8
	20–299 employees	43	25.6
	300–999 employees	1	0.6
Revenue of the previous year	Revenue less than RMB 3 million	115	68.5
	Revenue RMB 3–20 million	48	28.6
	Revenue RMB 20–40 million	4	2.4
	Revenue of more than RMB 40 million	1	0.6
Industry attributes	Information transmission, software and information technology services	74	44.0
	Scientific research and technology services	38	22.6
	Manufacturing	10	6.0
	Leasing and business services	12	7.1
	Culture, sports and entertainment	11	6.5
	Wholesale and retail	8	4.8
	Other industries	15	9.0
Whether there is overseas business	Export	11	6.5
	No export	157	93.5

### Measurement

The scales applied in this paper originates from the mature scales. This study adopts the back translation method to translate and back translate the items in the original scales and revised them with the actual situation. All scales adopt Likert 5-point Scale, ranging from “1 = totally disagree” to “5 = totally agree.”

1. The COVID-19 pandemic event’s strength. Based on the measurement scale developed by Morgeson et al. (2015), Morgeson (2005), and Morgeson and DeRue (2006), and revised in combination with the COVID-19 pandemic, it contains three dimensions: novelty, criticality and disruption with a total of 11 items. Novelty contains “there is a clear, known way for our company to respond to the COVID-19 pandemic event” and other three items. Criticality contains “the COVID-19 pandemic event is critical for the long-term success of our company’s innovation and development” and other two items. Disruption includes “the COVID-19 pandemic event disrupts our company’s value creation and acquisition ability to get its work done” and other three items. The items of novelty are the reverse scoring question.
2. *ETP.* Referring to the measurement scale of Lebel (2016), *ETP* includes “the COVID-19 pandemic event causes the economic downturn that would negatively impact our organization” and other four items.
3. *EPP.* Referring to Chen (2008) and Xu and Jia (2022), *PP* is measured by whether the actual performance is expected to be lower than the same period of the previous years. This paper makes use of the virtual variable (0–1) to measure *EPP*. If the actual performance of the enterprise is lower than the same period of the previous years, the entrepreneur will have a high *PP.* The assignment is 1. Otherwise, the assignment is 0.
4. *EA*. Referring to the scale of Lambert et al. (2014), anxiety is measured by three items: anxious, nervous, and worried. These three items are used to measure *EA*.

In addition, the study also takes the age of enterprises, number of employees, business income, industry attribute, and presence of overseas business as control variables. See Table 2 for the specific items of each scale.

**Table 2:** Measurement items and the reliability and validity of variables.

**Variable**	**Dimension**	**Item**	**EFA factor loading**	**CFA factor loading**	**Total variance explained**	**CR**	**Cronbach’s α**
*COVID-19 pandemic event’s strength* (KMO = 0.871)	*Novelty*	There is a clear, known way for our company to respond to the COVID-19 pandemic event®	0.898	0.904	30.222%	0.926	0.924
		There is an understandable sequence of steps that can be followed in responding for our company to the COVID-19 pandemic event®	0.920	0.937			
		Our company can rely on established procedures and practices in responding to The COVID-19 pandemic event®	0.887	0.830			
		our company had rules, procedures, or guidelines to follow when the COVID-19 pandemic event occurred®	0.842	0.803			
	*Criticality*	The COVID-19 pandemic event is critical for the long-term success of our company’s innovation and development	0.689	0.779	58.444%	0.877	0.872
		The COVID-19 pandemic event is of a priority to our company’s innovation and development	0.850	0.835			
		COVID-19 pandemic is an important event for our company’s innovation and development	0.775	0.900			
	*Disruption*	The COVID-19 pandemic event disrupts our company’s value creation and acquisition ability to get its work done	0.766	0.793	78.768	0.886	0.883
		The COVID-19 pandemic event causes our company to stop and think about how to respond	0.803	0.813			
		The COVID-19 pandemic event alters our company’s normal way of responding	0.856	0.861			
		The COVID-19 pandemic event requires our company to change the way it does its work	0.827	0.779			
*ETP*(KMO = 0.768)		The COVID-19 pandemic event causes the economic downturn that would negatively impact our organization	0.822	0.772	59.525%	0.827	0.823
		Affected by COVID-19 event, our organization would lose (part) sales or revenue	0.765	0.783			
		Affected by COVID-19 event, there would be layoffs at our organization	0.694	0.541			
		Affected by COVID-19 event, our organization would lose business to a competitor	0.731	0.579			
		The COVID-19 pandemic event causes an industry downturn that would negatively impact our organization	0.837	0.798			
*EA* (KMO = 0.753)		Anxious	0.933	0.910	89.563%	0.942	0.941
		Nervous	0.962	0.964			
		Worried	0.944	0.882			

^®^Refers to the reverse question.

### Data analysis tools and techniques

This paper makes use of SPSS 22.0 and AMOS 25.0 for data analysis. First, SPSS 22.0 is used for exploratory factor analysis and reliability analysis, and then AMOS 25.0 is for confirmatory factor analysis. On this basis, SPSS 22.0 is applied for descriptive statistics and correlation analysis, model 4 in the PROCESS for SPSS is for mediating effect test, and model 59 in the PROCESS for SPSS is utilized for the total effect moderation model test.

### Testing of reliability and validity

The Cronbach’s α of each variable and the combined reliability are shown in Table 2. The Cronbach’s α of novelty, criticality and disruption of the COVID-19 pandemic event are 0.924, 0.872 and 0.883 respectively, and the combined reliability are 0.926, 0.877 and 0.886 respectively, indicating that the scale has good internal consistency and reliability. The Cronbach’s α of *ETP* is 0.823 and the combined reliability is 0.827, which suggests the scale has good internal consistency and reliability. The Cronbach’s α of *EA* is 0.941, and the combined reliability is 0.942, which evinces that the scale has good internal consistency and reliability.

In terms of validity, exploratory factor analysis (EFA) is used to test the aggregation validity of each scale, and the results are shown in Table 2. The KMO value of EFA for the COVID-19 pandemic event’s strength is 0.871, which manifests that the scale is suitable for EFA. Then the maximum variance method is rotated. The results reflect that the items could be clustered into three dimensions: novelty, criticality and disruption. The total variance of the cumulative explanation is 78.768%, which indicates that the scale has good validity. The KMO value of EFA for the *ETP* is 0.768, showing that it is suitable for EFA. Then the maximum variance method is rotated. One factor is extracted from the result, and the total variance of cumulative explanation is 59.525%, which evinces that the scale has good aggregation validity. The KMO value of EFA for the *EA* is 0.753, meaning that it is suitable for EFA. Then the maximum variance method is rotated. One factor is extracted from the result, and the total variance of cumulative explanation is89.563%, which reveals that the scale has good convergent validity.

On the basis of EFA, confirmatory factor analysis (CFA) is used to test the discriminant validity of the scales. The results are shown in Table 3. The fitting degree of the five-factor model (χ^2^/*df* = 1.873 < 3, *RMR* = 0.050, *RMSEA* = 0.072 < 0.08, *CFI* > 0.9, *TLI* > 0.9, *IFI* > 0.9, PCFI >0.5) is better than that of the single-factor model and other competitive models, which shows that there is a good distinction between event’s novelty, criticality and disruption, *ETP* and *EA*.

**Table 3:** Confirmatory factor analysis results.

**variables**	***χ***^***2***^	***df***	***χ***^***2***^***/df***	***RMR***	***RMSEA***	***CFI***	***TLI***	***IFI***	***PCFI***
5-factor model	265.969	142	1.873	0.050	0.072	0.947	0.936	0.947	0.786
5-factor model + common method factor	221.531	123	1.801	0.050	0.069	0.958	0.941	0.958	0.689
4-factor model	468.305	146	3.208	0.097	0.115	0.861	0.838	0.863	0.735
3-factor model	830.472	148	5.611	0.103	0.166	0.706	0.661	0.709	0.611
2-factor model	1004.999	150	6.700	0.129	0.185	0.632	0.581	0.635	0.554
1-factor model	1339.904	152	8.815	0.120	0.216	0.489	0.425	0.493	0.434

5-factor model: novelty, criticality, disruption, threat, Anxiety. 4-factor model: novelty, criticality, disruption, threat + Anxiety. 3-factor model: novelty + criticality + disruption, threat, Anxiety. 2-factor model: novelty + criticality + disruption, threat + Anxiety. 1-factor model: novelty + criticality + disruption + threat + Anxiety.

### Common method bias test

The questionnaire items are filled by entrepreneurs, so there may be common variance in the process of data collection. This paper firstly utilizes Harman’s one-factor method to test the common method bias, and all items are put together to perform factor analysis. As the results show, factor 1 occupies 24.553% of the variance, less than 40% and less than half of the total variance of 72.978%, indicating that there was no serious common method bias in this paper. At the same time, the common method factor is introduced into the CFA, and the fitting index after controlling the common method factor is shown in Table 3. The results show that the common method factor (*RMSEA* = 0.072, *CFI* = 0.947, *IFI* = 0.947) is added to the five-factor model (*RMSEA* = 0.069, *CFI* = 0.958, *IFI* = 0.958), and the improvement of model fit index is very small, evincing that the problem of common variance among the variables is not salient.

## Empirical results

### Descriptive statistics and correlation analysis

The results of descriptive statistics and correlation analysis are shown in Table 4. The results reflect that the COVID-19 pandemic is positively correlated with *ETP* (*r* = 0.629, *p* < 0.001) and *EA* (*r* = 0.387, *p* < 0.001). Meanwhile, there is a positive correlation between *ETP* and *EA* (*r* = 0.539, *p* < 0.001), which is significant at the 0.001 level respectively, initially supporting the research hypothesis of this paper. In addition, the AVE square root of the latent variables is larger than the correlation coefficient between the variables, which shows that the core variables has higher discriminant validity. In order to further test the hypothesis proposed in this paper, the following regression analysis is introduced.

**Table 4:** Means, standard deviations and correlations coefficients.

**Variables**	**1**	**2**	**3**	**4**
1 COVID-19 pandemic	**0.841**			
2 Entrepreneur Performance Pressure	0.367^***^	**–**		
3 Entrepreneur Threat Perception	0.629^***^	0.257^***^	**0.703**	
4 Entrepreneur Anxiety	0.387^***^	0.157^*^	0.539^***^	**0.919**
Mean	3.024	0.792	3.226	3.635
SD	0.460	0.407	0.738	0.802

The bold part in the table is the AVE square root of each latent variable.

*** *p* < 0.001;

* *p *< 0.050.

### Hypothesis test

Hierarchical regression is applied firstly in this paper to analyze the direct effect of the COVID-19 pandemic on *EA* and the mediating effect of *ETP.* Then the total effect moderation model proposed by Edwards and Lambert (2007) is used to test the moderating effect of *EPP*, and the results are shown in Table 5. The empirical test result of the relationship between the COVID-19 pandemic and *EA* is shown in M2. The result shows that the COVID-19 pandemic has a significantly positive effect on *EA* (*β* = 0.682, *p* < 0.001). The hypothesis 1 is supported.

**Table 5:** Regression results of COVID-19 pandemic on *EA*.

**Variables**	***EA***		***ETP***		***EA***	
	**M1**	**M2**	**M3**	**M4**	**M5**	**M6**
Constant	3.734	1.707	0.376	0.495	1.506	1.876
Enterprise age	−0.005	−0.026	0.017	0.017	−0.035	−0.026
Number of employees	−0.015	−0.087	−0.096	−0.095	−0.036	−0.029
Revenue of the previous year	−0.031	0.057	−0.036	−0.036	0.076	0.046
Dummy variable_ Manufacturing	−0.430	−0.152	−0.157	−0.151	−0.068	−0.135
Dummy variable_ overseas	0.104	−0.014	0.018	0.008	−0.023	−0.026
COVID-19 pandemic		0.682^***^	0.983^***^	0.926^***^	0.158	−0.293
*ETP*					0.534^***^	0.829^***^
*EPP*				−0.166		−0.621^***^
COVID-19 pandemic × *EPP*				0.074		0.663^*^
*ETP* × *EPP*						−0.417^*^
*R*	0.134	0.398	0.636	0.636	0.550	0.569
*R* ^2^	0.018	0.159	0.404	0.405	0.302	0.324
*F*	0.591	5.063^***^	18.181^***^	13.521^***^	9.907^***^	7.517^***^

The coefficient is a non-standardized regression coefficient.

*** *p *< 0.001;

* *p *< 0.050.

M2, M3 and M5 are used to test the mediating effect of *ETP*. The results show that the COVID-19 pandemic has a significantly positive effect on *ETP* (*β* = 0.983, *p* < 0.001), and *ETP* also has a significantly positive effect on *EA* (*β* = 0.534, *p* < 0.001). The regression coefficient of the COVID-19 pandemic on *EA* became smaller, and the significance changes from being significant to non-significant. Furthermore, bootstrap test results suggest that the mediating effect of *ETP* is 0.524 with 95% confidence interval [0.293, 0.769] and does not contain zero. Therefore, *ETP* plays a significantly mediating role between the COVID-19 pandemic and *EA*, and the hypothesis 2 is supported.

The results of moderating effect analysis of *EPP* can be found in M4 and M6. The results show that the interaction between *EPP* and the COVID-19 pandemic positively affects *EA* (*β* = 0.663, *p* < 0.05). That is, the greater *EPP* is, the stronger the positive impact of the COVID-19 pandemic on *EA* will be, and the hypothesis 3a is supported. The interaction item between *EPP* and the COVID-19 pandemic has no significant effect on *ETP* (*β* = 0.074, *p* > 0.05), so the hypothesis 3b is not supported. The interaction item between *EPP* and *ETP* negatively affects *EA* (*β* = −0.417, *p* < 0.05). That is, the stronger *EPP* is, the weaker the positive effect of *ETP* on *EA* will be, and the hypothesis 3c is supported.

**Figure 2:**
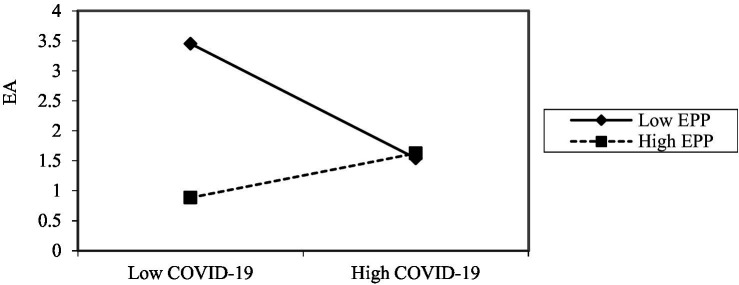
The moderating effect of *EPP* between COVID-19 pandemic and *EA.*

**Figure 3:**
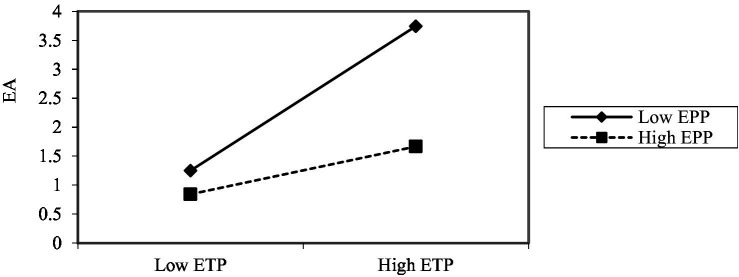
The moderating effect of *EPP* between *ETP* and *EA.*

In order to present the moderating effect of *EPP* much clearer, a simple slope diagram is applied to plot the moderating effects of *EPP* on the relationship between the COVID-19 pandemic and *EA*, *ETP* and *EA* respectively, as shown in Figure 2, 3. According to Figure 2, it can be seen that under high *EPP*, the COVID-19 pandemic will increase *EA*, while it is negatively related to *EA* with low *EPP.* The reason may lie in that when the company has no performance decline (low *PP*), while its competitors’ performance decline, the company has advantage under the circumstances, and thus reducing anxiety. By virtue of Figure 3, it reveals that under high *PP*, the positive influence of *ETP* on *EA* is weak, while under low *PP*, the positive effect of the *ETP* on *EA* is more conspicuous.

## Discussion

### Results discussion

From the perspective of the COVID-19 pandemic event’s strength, based on the cognitive appraisal theory and the event system theory, this paper performs an in-depth and far-reaching study of the COVID-19 pandemic’s influence on entrepreneurs. According to the empirical analysis to the 168 entrepreneurs of *EFs* from the science and technology parks in Guangdong Province, China, the following results are obtained.

First, the COVID-19 pandemic event’s strength has a positive effect on *EA*. Given the novelty of the COVID-19, with the sudden outbreak of it, entrepreneurs are lack of a well-established procedure and process to cope with the sudden changes, resulting in uncertainty about their future goals. From the perspective of the disruption, the COVID-19 pandemic has an impact on all industries and sectors, which leads to declines in sales, market share and profits, and exacerbates the financial burden and uncertainty of entrepreneurs, and thus intensifying the anxiety of entrepreneurs. From the perspective of the criticality, the long-term impact of the COVID-19 pandemic will remain unknown in the future (Korsgaard et al., 2020), bringing uncertainty to entrepreneurs and threatening the realization of their goals and long-term development, further increasing their anxiety. Similar to the existing findings that “the COVID-19 pandemic have a negative impact on the entrepreneurs’ psychology of SMEs” (Lathabhavan, 2022a; Sornsenee et al., 2022). This article finds that the COVID-19 pandemic causes *EA* of *EF*s. However, the existing research puts high premium on entrepreneurs to explore the COVID-19 pandemic’s negative impact, but ignores the events’ attributes, that is, from the intrinsic characteristics of the event to explore the COVID-19 pandemic’s impact on entrepreneurs’ psychological mechanism. Considering the intrinsic characteristics of the COVID-19 pandemic including the novelty, the disruption and the criticality, will help to strengthen the explanation of how the COVID-19 pandemic affects entrepreneurs. More importantly, from a micro-perspective, this study examines the impact of the COVID-19 pandemic on entrepreneurs and extends the application of event system theory in the field of entrepreneurs’ emotions.

Second, *ETP* plays a significant mediating role between the COVID-19 pandemic and *EA*. Based on the cognitive appraisal theory, this study puts the COVID-19 pandemic on individuals into a process of “external stimulus--cognitive appraisal--emotional response,” and the mediating role of *ETP* between the COVID-19 pandemic and *EA* is analyzed and verified. This conclusion shows that the *EA* mainly comes from the cognitive appraisal process brought from the external threat of stimulus events. When individuals perceive the COVID-19 pandemic threat is greater, they will show a higher level of psychological anxiety. Most previous studies have regarded *TP* as an antecedent variable of anxiety (Cypryanska and Nezlek, 2020; Paredes et al., 2021). On this basis, in view of the impact of the COVID-19 pandemic, this paper takes the COVID-19 pandemic as the antecedent variable of *ETP*, and regards *ETP* as a mediator variable based on the cognitive appraisal theory. Previous studies explore the impact of COVID-19 pandemic on individual anxiety from the perspective of job insecurity (Lin et al., 2021) and efficacy (Zhang et al., 2022), while this study provides a new theoretical insight and mechanism for the research of the COVID-19 pandemic and individual emotion through the verification of *TP* mechanism, that is, to understand the influential mechanism of the COVID-19 pandemic on anxiety from the perspective of *TP*, which enriches the research results of the influential mechanism of the COVID-19 pandemic on entrepreneur’s emotion.

Third, *EPP* positively moderates the relationship between the COVID-19 pandemic event’s strength and *EA*, and it negatively moderates the relationship between *ETP* and *EA*. However, the moderating effect on the relationship between the COVID-19 pandemic event’s strength and *ETP* is not significant. The results illuminate that *EPP* has a significant positive moderating effect between the COVID-19 pandemic event’s strength and *EA*. For entrepreneurs with high *PP*, under the influence of the COVID-19 pandemic, the gap between the expected performance this year and that of the same period in the previous years is larger. Furthermore, they will face more potential losses, which in turn brings about higher levels of anxiety. Besides, there is no significant moderating effect between the COVID-19 pandemic and *ETP*, and that is because the level of internal performance pressure may not change the objective threat incurred by external crisis. Entrepreneurial stress stems from external uncertainty (Rauch et al., 2018), which in turn threatens individuals. For entrepreneurs, however high nor low *PP* it is, it cannot change the uncertainty caused by the COVID-19 pandemic. In addition, other variables may also modulate the relationship between the COVID-19 pandemic and *TP*, such as the entrepreneurs’ characteristics and risk perception. Entrepreneurs with different risk perceptions tend to have different *TP*. Eventually, there is a significant negative moderating effect of *EPP* on *ETP* and *EA*. High-pressure entrepreneurs are inclined to appear stronger crisis awareness, opportunity recognition, and crisis coping, often regarding threats as external challenges, which can alleviate the anxiety caused by external threats to some extent. On the basis of Xu and Jia’s (2022) model of *PP* moderating COVID-19 pandemic and entrepreneur’ psychological well-being, this study further investigates the effect boundary of both the COVID-19 pandemic on *ETP* and *ETP* on *EA*, broadening the model proposed by Xu and Jia (2022) and revealing the influential mechanism of *PP* on *EA* under the COVID-19 pandemic as well. What is more, the study finds that individual pressure can affect their cognitive appraisal, which broadens the theoretical model of cognitive appraisal proposed by Skinner and Brewer (2002) from the perspective of pressure. It means that future research should take the pressure influential mechanism into consideration in the process of exploring “external stimulus--cognitive appraisal--emotional response.”

### Theoretical implications

Based on the above research findings, this paper has the following theoretical implications.

First, this paper explores the impact of the COVID-19 pandemic event’s strength on entrepreneurial anxiety. Similar to the existing findings that “the COVID-19 pandemic has a negative impact on the entrepreneurs’ psychology of SMEs” (Sornsenee et al., 2022; Lathabhavan, 2022a). This paper finds that the COVID-19 pandemic causes *EA* of *EF*s. However, the existing research puts high premium on entrepreneurs to explore the COVID-19 pandemic’s negative impact, but ignores the events’ attributes. That is, from the intrinsic characteristics of the event to explore the influential mechanism of the COVID-19 pandemic impacting entrepreneurs’ psychology. Considering the intrinsic characteristics of the COVID-19 pandemic including the novelty, the disruption and the criticality, this study will help to strengthen the explanation of how the COVID-19 pandemic affects entrepreneurs. More importantly, from a micro-perspective, this study examines the impact of the COVID-19 pandemic on entrepreneurs and extends the application of event system theory in the field of entrepreneurs’ emotions.

Second, this paper introduces *TP* as a mediating variable of the COVID-19 pandemic event’s strength affecting entrepreneurs’ anxiety. Most previous studies have regarded *TP* as an antecedent variable of anxiety (Cypryanska and Nezlek, 2020; Paredes et al., 2021). On this basis, in view of the impact of the COVID-19 pandemic, this paper takes the COVID-19 pandemic as the antecedent variable of *ETP*, and regards *ETP* as a mediator variable based on the cognitive appraisal theory. Previous studies explore the impact of COVID-19 pandemic on individual anxiety from the perspective of job insecurity (Lin et al., 2021) and efficacy (Zhang et al., 2022), while this study provides a new theoretical insight and mechanism for the research of the COVID-19 pandemic and individual emotion through the verification of *TP* mechanism. That is, to understand the influential mechanism of the COVID-19 pandemic on anxiety from the perspective of *TP*, which enriches the research results of the influential mechanism of the COVID-19 pandemic on entrepreneur’s emotion.

Finally, on the basis of Xu and Jia’s (2022) model of *PP* moderating COVID-19 pandemic and entrepreneur’ psychological well-being, this study further investigates the effect boundary of both the COVID-19 pandemic on *ETP* and *ETP* on *EA*, broadening the model proposed by Xu and Jia (2022) and revealing the influential mechanism of *PP* on *EA* under the COVID-19 pandemic as well. What is more, the study finds that individual pressure can affect their cognitive appraisal, which broadens the theoretical model of cognitive appraisal proposed by Skinner and Brewer (2002) from the perspective of pressure. It means that future research should take the pressure influential mechanism into consideration in the process of exploring “external stimulus--cognitive appraisal--emotional response.”

### Practical implications

Based on the above research findings, this paper puts forward the following practical implications.

First, in view of the adverse impact of the COVID-19 pandemic, the science and technology parks should develop contingency plans for major crises such as the COVID-19 pandemic. The science and technology parks should promote the digital transformation of *EFs* in order to reduce the restrictions on business operations caused by the pandemic and enhance entrepreneurs’ confidence in coping with major crisis. Through the above measures, entrepreneurs’ perception of the intensity of major crisis events such as the COVID-19 epidemic can be reduced, thus soothing entrepreneurs’ psychological anxiety. In addition, enterprises should establish clear and executable working procedures and guidelines during the pandemic to decrease the threat and negative impact of COVID-19 pandemic to entrepreneurs and *EFs*, helping them quickly return to normal operations.

Second, given that *EPP* is positively moderating the relationship between the COVID-19 pandemic event’s strength and *EA,* entrepreneurs should fully understand the negative impact of the COVID-19 pandemic and other major crises, and appropriately adjust their firm performance targets to reduce *PP* caused by high targets. In addition, the science and technology parks should constantly enhance the entrepreneurs’ crisis awareness and the ability of identifying and responding to crisis, and of recognizing and grabbing opportunities. The last but not least, to organize concentrated capacity training to deepen entrepreneurs’ understanding of major crises such as the COVID-19 pandemic and instruct them how to identify and exploit opportunities under major crises, which will facilitate them to cope with sudden major crises better in the future as well.

Third, in view of the mediating role of *TP* between the COVID-19 pandemic and *EA*, in order to alleviate *EA* and maintain entrepreneurs a good psychological state, it is better to minimize the external *ETP*. Entrepreneurs should spare no efforts to obtaining information for a correct understanding of the crisis, thus reducing the threat of information asymmetry and uncertainty. At the same time they need to treat pandemic threat in a correct way. By viewing perceived external crises as external challenges, they can better improve their emotional state, and thus face crises and challenges bravely and identify opportunities positively, ultimately promoting the long-term growth of *EFs*.

### Limitations and future research

Although the research findings of this paper have some theoretical and practical implications, but there are still some limitations. First and foremost, as it is not quite easy for entrepreneurs to carry out the longitudinal questionnaire survey, this paper uses cross-sectional data to study the impact of the COVID-19 pandemic on *EA*, changes in core variables are difficult to be measured. Therefore, future research should adopt longitudinal follow-up study to further test the cause and effect of research conclusions. Secondly, based on the cognitive appraisal theory and the event system theory, this paper explores the influential mechanism of the COVID-19 pandemic event’s strength affecting the *EA* through *ETP*, and introduce *EPP* as a moderator to explore the effecting boundary of the COVID-19 pandemic on *EA*, obtaining some meaningful conclusions indeed. However, the relationship between the COVID-19 pandemic and *EA* is not only influenced by *EPP* and *EPT*, but also by other individual characteristics such as entrepreneur personality. Therefore, the impact of different individual characteristics on the relationship between the COVID-19 pandemic and entrepreneurs’ negative emotions should be taken into account in the future studies, further exploring the “black box” of negative events on entrepreneurs’ negative emotions. In the end, this paper uses event’s strength to measure the impact of the COVID-19 pandemic. Nevertheless, event systems theory also focuses on explaining the effects of events on organizations and individuals from temporal and spatial attributes (Morgeson et al., 2015), and subsequent research can expand the event measurement dimension, from the time and space attributes to measure the COVID-19 pandemic, further delving into its impact on entrepreneurs.

## Conclusion

This study investigates the mediating effect of *ETP* and the moderating effect of *EPP* by exploring the influential mechanism of the COVID-19 pandemic influencing *EA*. Through the empirical study, this paper finds that the COVID-19 pandemic significantly and positively affects *EA*, and it will facilitate *ETP*, then indirectly affecting *EA*. In other words, *ETP* plays a mediating role between COVID-19 pandemic and *EA*. In addition, this study also finds that the positive effect of COVID-19 pandemic on *EA* is enhanced by *EPP*, while the positive effect of *ETP* on *EA* is weakened by *EPP*. This study will be favorable for researchers and managers to further understand the potential relationship and mechanism between the COVID-19 pandemic and *EA* from the event system theory and cognitive appraisal theory. In a nutshell, the findings will enrich the research results in the field of the COVID-19 pandemic and entrepreneurs’ emotion, providing theoretical reference for timely intervention of entrepreneurs’ mental health under sudden crisis, which will facilitate the healthy development of entrepreneurial firms, the stability of social employment and the economic recovery.

## Data availability statement

The original contributions presented in the study are included in the article/supplementary material, further inquiries can be directed to the corresponding author.

## Ethics statement

Ethical review and approval were not required for the study on human participants in accordance with the local legislation and institutional requirements. The participants have provided their written informed consent to participate in this study.

## Author contributions

All authors listed have made a substantial, direct, and intellectual contribution to the work and approved it for publication.

## Funding

This research is supported by the National Natural Science Foundation of China (71802062), the Science and Technology Planning Project of Guangdong (2020B1010010013 and 2018A070712042), the “13th five year plan” for the Development of Philosophy and Social Sciences in Guangzhou (2020GZGJ161).

## Conflict of interest

The authors declare that the research was conducted in the absence of any commercial or financial relationships that could be construed as a potential conflict of interest.

## Publisher’s note

All claims expressed in this article are solely those of the authors and do not necessarily represent those of their affiliated organizations, or those of the publisher, the editors and the reviewers. Any product that may be evaluated in this article, or claim that may be made by its manufacturer, is not guaranteed or endorsed by the publisher.

## References

[ref1] Ahorsu D. K. Lin C. Y. Imani V. Saffari M. Griffiths M. D. Pakpour A. H. (2020). The fear of COVID-19 scale: development and initial validation. Int. J. Ment. Health Ad. 20, 1537–1545. doi: 10.1007/s11469-020-00270-8, PMID: 32226353 PMC7100496

[ref2] Alim M. A. Tan K. L. Jee T. W. Voon B. H. Hossain M. J. Mia M. U. (2022). To explain and to predict: analysis of opportunity recognition on the relationship between personal factors, environmental factors and entrepreneurs’ performance. Asia-Pac. J. Bus. Adm. doi: 10.1108/APJBA-09-2021-0475

[ref3] Arnold M. B. (1960). Emotion and personality: Psychological aspects. New York: Columbia University Press.

[ref4] Aujirapongpan S. Ru-Zhe J. Jutidharabongse J. (2020). Strategic intuition capability toward performance of entrepreneurs: evidence from Thailand. J. Asian Financ. Econ. 7, 465–473. doi: 10.13106/jafeb.2020.vol7.no6.465

[ref5] Backman M. Hagen J. Kekezi O. Naldi L. Wallin T. (2021). In the eye of the storm: entrepreneurs and well-being during the COVID-19 crisis. Entrep. Theory Pract. 10422587211057028:104225872110570. doi: 10.1177/10422587211057028

[ref6] Bartik A. W. Bertrand M. Cullen Z. Glaeser E. L. Luca M. Stanton C. (2020). The impact of COVID-19 on small business outcomes and expectations. P. Natl. Acad. Sci. USA. 117, 17656–17666. doi: 10.1073/pnas.2006991117, PMID: 32651281 PMC7395529

[ref7] Beleska-Spasova E. Glaister K. W. Stride C. (2012). Resource determinants of strategy and performance: the case of British exporters. J. World Bus. 47, 635–647. doi: 10.1016/j.jwb.2011.09.001

[ref8] Benedetti-Fasil C. Sedlacek P. Sterk V. (2022). Startups and employment following the COVID-19 pandemic: a calculator. Econ. Policy 37, 507–533. doi: 10.1093/epolic/eiac028

[ref9] Byrd K. Her E. Fan A. Almanza B. Liu Y. R. Leitch S. (2021). Restaurants and COVID-19: what are consumers' risk perceptions about restaurant food and its packaging during the pandemic? Int. J. Hosp. Manag. 94:102821. doi: 10.1016/j.ijhm.2020.102821, PMID: 34866742 PMC8631525

[ref10] Cacciotti G. Hayton J. C. (2015). Fear and entrepreneurship: a review and research agenda. Int. J. Manag. Rev. 17, 165–190. doi: 10.1111/ijmr.12052

[ref11] Chen W. R. (2008). Determinants of firms' backward- and forward-looking R&D search behavior. Organ. Sci. 19, 609–622. doi: 10.1287/orsc.1070.0320

[ref12] Chhatwani M. Mishra S. K. Varma A. Rai H. (2022). Psychological resilience and business survival chances: a study of small firms in the USA during COVID-19. J. Bus. Res. 142, 277–286. doi: 10.1016/j.jbusres.2021.12.048

[ref13] Costa C. Teodoro M. Mento C. Giambò F. Vitello C. Italia S.. (2022). Work performance, mood and sleep alterations in home office workers during the COVID-19 pandemic. Int. J. Env. Res. Pub. He. 19:990. doi: 10.3390/ijerph19041990, PMID: 35206177 PMC8871883

[ref14] Cuerdo-Vilches T. Navas-Martin M. A. March S. Oteiza I. (2021). Adequacy of telework spaces in homes during the lockdown in Madrid, according to socioeconomic factors and home features. Sustain. Cities Soc. 75:103262. doi: 10.1016/j.scs.2021.103262

[ref15] Cypryanska M. Nezlek J. B. (2020). Anxiety as a mediator of relationships between perceptions of the threat of COVID-19 and coping behaviors during the onset of the pandemic in Poland. PLoS One 15:e0241464. doi: 10.1371/journal.pone.0241464, PMID: 33125435 PMC7599044

[ref16] Damodaran A. (2009). Valuing young, startup and growth companies: estimation issues and valuation challenges. SSRN Electron. J. Available at: https://ssrn.com/abstract=1418687 (Accessed June 12, 2021).

[ref17] Dragan G. B. Bleoju G. Capatina A. Woodside A. (2022). European tech startups’ responses to the COVID-19 pandemic: integrating McKinsey’s 5R’s paradigm and the Newtonian gravitational field. Manage. Decis. 60, 2615–2636. doi: 10.1108/md-11-2021-1428

[ref18] Edwards J. R. Lambert L. S. (2007). Methods for integrating moderation and mediation: a general analytical framework using moderated path analysis. Psychol. Methods 12, 1–22. doi: 10.1037/1082-989X.12.1.1, PMID: 17402809

[ref19] Ellis L. Ward P. (2022). The effect of a high-pressure protocol on penalty shooting performance, psychological, and psychophysiological response in professional football: a mixed methods study. J Sport Sci. 40, 3–15. doi: 10.1080/02640414.2021.1957344, PMID: 34847831

[ref20] Frijda N. H. (1987). Emotion, cognitive structure, and action tendency. Cogn. Emot. 1, 115–143. doi: 10.1080/02699938708408043

[ref21] Frijda N. H. Kuipers P. ter Sehure E. (1989). Relations among emotion, appraisal, and emotional action readiness. J. Pers. Soc. Psychol. 57, 212–228. doi: 10.1037/0022-3514.57.2.212

[ref22] Garcia-Vidal G. Guzman-Vilar L. Sanchez-Rodriguez A. Martinez-Vivar R. Perez-Campdesuner R. Uset-Ruiz F. (2020). Facing post COVID-19 era, what is really important for Ecuadorian SMEs? Int. J. Eng. Bus. Manag. 12:184797902097194. doi: 10.1177/1847979020971944

[ref23] Hernandez-Sanchez B. R. Cardella G. M. Sanchez-Garcia J. C. (2020). Psychological factors that lessen the impact of COVID-19 on the self-employment intention of business administration and economics' students from Latin America. Int. J. Env. Res. Pub. He. 17:5293. doi: 10.3390/ijerph17155293, PMID: 32708034 PMC7432839

[ref24] Hoang H. Nguyen C. Nguyen D. K. (2022). Corporate immunity, national culture and stock returns: startups amid the COVID-19 pandemic. Int. Rev. Financ. Anal. 79:101975. doi: 10.1016/j.irfa.2021.101975 PMC8590515 36530769

[ref25] Hosany S. (2012). Appraisal determinants of tourist emotional responses. J. Travel Res. 51, 303–314. doi: 10.1177/0047287511410320

[ref26] Jang S. Kim J. Kim J. Kim S. (2021). Spatial and experimental analysis of peer-to-peer accommodation consumption during COVID-19. J. Destin. Mark. Manage. 20:100563. doi: 10.1016/j.jdmm.2021.100563

[ref27] Jhajharia G. Sharma G. (2021). Impact of COVID-19 pandemic on entrepreneurial actions in India: a thematic analysis. Jims8m-J. Indian Mama. 26, 35–47. doi: 10.5958/0973-9343.2021.00017.X

[ref28] Johns G. (2017). Reflections on the 2016 decade award: incorporating context in organizational research. Acad. Manag. Rev. 42, 577–595. doi: 10.5465/amr.2017.0044

[ref29] Kim J. Park J. Kim S. Lee D. C. Sigala M. (2021). COVID-19 restrictions and variety seeking in travel choices and actions: the moderating effects of previous experience and crowding. J. Travel Res. 61, 1648–1665. doi: 10.1177/00472875211037744

[ref30] Korsgaard S. Hurt R. A. Townsend D. M. Ingstrup M. B. (2020). COVID-19 and the importance of space in entrepreneurship research and policy. Int. Small Bus. J. Res. Entrep. 38, 697–710. doi: 10.1177/0266242620963942, PMID: 35125602 PMC7565246

[ref31] Kuckertz A. Brändle L. Gaudig A. Hinderer S. Reyes C. A. M. Prochotta A.. (2020). Startups in times of crisis – a rapid response to the COVID-19 pandemic. J. Bus. Ventur. Insights 13:e00169. doi: 10.1016/j.jbvi.2020.e00169

[ref32] Lambert A. J. Eadeh F. R. Peak S. A. Scherer L. D. Schott J. P. Slochower J. M. (2014). Toward a greater understanding of the emotional dynamics of the mortality salience manipulation: revisiting the "affect-free" claim of terror management research. J. Pers. Soc. Psychol. 106, 655–678. doi: 10.1037/a0036353, PMID: 24749817

[ref33] Lathabhavan R. (2022a). COVID-19 and mental health concerns among business owners: a cross-sectional study from India. Int. J. Ment. Health Ad. 1–11. doi: 10.1007/s11469-022-00824-y, PMID: 35465028 PMC9017730

[ref34] Lathabhavan R. (2022b). Fear of COVID-19, psychological distress, well-being and life satisfaction: a comparative study on first and second waves of COVID-19 among college students in India. Curr. Psychol. 1–8. doi: 10.1007/s12144-022-03207-7, PMID: 35637761 PMC9132172

[ref35] Lazarus R. S. (1991). Progress on a cognitive-motivational-relational theory of emotion. Am. Psychol. 46, 819–834. doi: 10.1037/0003-066X.46.8.819, PMID: 1928936

[ref36] Lebel R. D. (2016). Overcoming the fear factor: how perceptions of supervisor openness lead employees to speak up when fearing external threat. Organ. Behav. Hum. Dec. 135, 10–21. doi: 10.1016/j.obhdp.2016.05.001

[ref37] Li Y. J. Chen H. C. Wei L. L. Wei L. Q. (2022). COVID-19 pandemic and SMEs performance decline: the mediating mole of management innovation and organizational resilience. Front. Public Health 10:944742. doi: 10.3389/fpubh.2022.944742, PMID: 35903388 PMC9315063

[ref38] Lin W. P. Shao Y. D. Li G. Q. Guo Y. R. Zhan X. J. (2021). The psychological implications of COVID-19 on employee job insecurity and its consequences: the mitigating role of organization adaptive practices. J. Appl. Psychol. 106, 317–329. doi: 10.1037/apl0000896, PMID: 33871269

[ref39] Liu B. J. Lu L. Zhang H. Liu C. J. (2021). Strategic choices for social responsibility of startups in China. Front. Psychol. 12:719454. doi: 10.3389/fpsyg.2021.719454, PMID: 34646207 PMC8503524

[ref40] Mamun M. A. Akter S. Hossain I. Faisal M. T. H. Rahman M. A. Arefin A.. (2020). Financial threat, hardship and distress predict depression, anxiety and stress among the unemployed youths: a bangladeshi multi-city study. J. Affect. Disorders. 276, 1149–1158. doi: 10.1016/j.jad.2020.06.075, PMID: 32791351

[ref41] Mckibbin W. Fernando R. (2021). The global macroeconomic impacts of COVID-19: seven scenarios. Asian Econ. Pap. 20, 1–30. doi: 10.1162/asep_a_00796

[ref42] Mesagno C. Mullane-Grant T. (2010). A comparison of different pre-performance routines as possible choking interventions. J. Appl. Sport Psychol. 22, 343–360. doi: 10.1080/10413200.2010.491780

[ref43] Meurer M. M. Waldkirch M. Schou P. K. Bucher E. L. Burmeister-Lamp K. (2022). Digital affordances: how entrepreneurs access support in online communities during the COVID-19 pandemic. Small Bus. Econ. 58, 637–663. doi: 10.1007/s11187-021-00540-2 PMC8503734 38624988

[ref44] Mitchell M. S. Greenbaum R. L. Vogel R. M. Mawritz M. B. Keating D. J. (2019). Can you handle the pressure? The effect of performance pressure on stress appraisals, self-regulation, and behavior. Acad. Manag. J. 62, 531–552. doi: 10.5465/amj.2016.0646

[ref45] Moral I. H. Rahaman M. S. Imran M. S. Rahman M. M. (2022). Mental health of hawkers during COVID-19: a marginal community in Bangladesh. J. Enterp. Communities. doi: 10.1108/JEC-01-2022-0006

[ref46] Morgeson F. P. (2005). The external leadership of self-managing teams: intervening in the context of novel and disruptive events. J. Appl. Psychol. 90, 497–508. doi: 10.1037/0021-9010.90.3.497, PMID: 15910145

[ref47] Morgeson F. P. DeRue D. S. (2006). Event criticality, urgency, and duration: understanding how events disrupt teams and influence team leader intervention. Leadership Quart. 17, 271–287. doi: 10.1016/j.leaqua.2006.02.006

[ref48] Morgeson F. P. Mitchell T. R. Liu D. (2015). Event system theory: an event-orientedapproach to the organizational sciences. Acad. Manag. Rev. 40, 515–537. doi: 10.5465/amr.2012.0099

[ref49] Mota R. D. Bueno A. Gonella J. D. L. Ganga G. M. D. Godinho M. Latan H. (2022). The effects of the COVID-19 crisis on startups' performance: the role of resilience. Manage. Decis. doi: 10.1108/MD-07-2021-0998

[ref50] Mustafa F. Khursheed A. Fatima M. Rao M. R. (2021). Exploring the impact of COVID-19 pandemic on women entrepreneurs in Pakistan. Int. J. Gend. Entrep. 13, 187–203. doi: 10.1108/IJGE-09-2020-0149

[ref51] Nummela N. Paavilainen-Mantymaki E. Harikkala-Laihinen R. Raitis J. (2020). When all doors close: implications of COVID-19 for cosmopolitan entrepreneurs. Int. Small Bus. J. 38, 711–717. doi: 10.1177/0266242620954127, PMID: 35125603 PMC7488169

[ref52] Oatley K. Johnson-Laird P. N. (2014). Cognitive approaches to emotions. Trends Cogn. Sci. 18, 134–140. doi: 10.1016/j.tics.2013.12.004 24389368

[ref53] OECD. (2020). Startups in the time of COVID-19: Facing the challenges, seizing the opportunities. Retrieved from https://www.oecd.org/coronavirus/policy-responses/start-ups-in-the-time-of-covid-19-facing-the-challenges-seizing-the-opportunities-87219267/ [Accessed May 13, 2020].

[ref54] Paredes M. R. Apaolaza V. Fernandez-Robin C. Hartmann P. Yanez-Martinez D. (2021). The impact of the COVID-19 pandemic on subjective mental well-being: the interplay of perceived threat, future anxiety and resilience. Pers. Indiv. Differ. 170:110455. doi: 10.1016/j.paid.2020.110455, PMID: 33071413 PMC7552984

[ref55] Qu S. W. Li T. H. Zhang M. (2022). Predictive factors of the entrepreneurial performance of undergraduates. Front. Psychol. 13:814759. doi: 10.3389/fpsyg.2022.814759, PMID: 35360614 PMC8963528

[ref56] Rahman M. M. Tabash M. I. Salamzadeh A. Abduli S. Rahaman M. S. (2002). Sampling techniques (probability) for quantitative social science researchers: a conceptual guidelines with examples. SEEU Review 17, 42–51. doi: 10.2478/seeur-2022-0023

[ref57] Ramadani V. Rahman M. M. Salamzadeh A. Rahaman M. S. Abazi-Alili H. (2022). Entrepreneurship education and graduates’ entrepreneurial intentions: does gender matter? A multi-group analysis using AMOS. Technol. Forecast. Soc. 180:121693. doi: 10.1016/j.techfore.2022.121693

[ref58] Rauch A. Fink M. Hatak I. (2018). Stress processes: an essential ingredient in the entrepreneurial process. Acad. Manage. Perspect. 32, 340–357. doi: 10.5465/amp.2016.0184

[ref59] Roseman I. J. (1996). Appraisal determinants of emotions: constructing a more accurate and comprehensive theory. Cogn. Emot. 10, 241–278. doi: 10.1080/026999396380240

[ref60] Roseman I. J. Spindel M. S. Jose P. E. (1990). Appraisals of emotion-eliciting events: testing a theory of discrete emotions. J. Pers. Soc. Psychol. 59, 899–915. doi: 10.1037/0022-3514.59.5.899

[ref61] Sarker M. R. Rahman S. M. A. Islam A. K. M. H. Bhuyan M. F. F. Supra S. E. Ali K.. (2022). Impact of COVID-19 on small- and medium-sized enterprises. Glob. Bus. Rev.:097215092210934. doi: 10.1177/09721509221093489

[ref62] Satici B. Gocet-Tekin E. Deniz M. E. Satici S. A. (2021). Adaptation of the fear of COVID-19 scale: its association with psychological distress and life satisfaction in Turkey. Int. J. Ment. Health Ad. 19, 1980–1988. doi: 10.1007/s11469-020-00294-0, PMID: 32395095 PMC7207987

[ref63] Shagirbasha S. Sivakumaran B. (2021). Cognitive appraisal, emotional labor and organizational citizenship behavior: evidence from hotel industry. J. Hosp. Tour. Manag. 48, 582–592. doi: 10.1016/j.jhtm.2021.08.016

[ref64] Shah S. H. A. Haider A. Jindong J. Mumtaz A. Rafiq N. (2022). The impact of job stress and state anger on turnover intention among nurses during COVID-19: the mediating role of emotional exhaustion. Front. Psychol. 12:810378. doi: 10.3389/fpsyg.2021.810378, PMID: 35222162 PMC8863937

[ref65] Shen H. Fu M. Y. Pan H. Y. Yu Z. F. Chen Y. Q. (2020). The impact of the COVID-19 pandemic on firm performance. Emerg. Mark. Financ. Tr. 56, 2213–2230. doi: 10.1080/1540496X.2020.1785863

[ref66] Skinner N. Brewer N. (2002). The dynamics of threat and challenge appraisals prior to stressful achievement events. J. Pers. Soc. Psychol. 83, 678–692. doi: 10.1037/0022-3514.83.3.678, PMID: 12219862

[ref67] Smith C. A. Ellsworth P. C. (1985). Patterns of cognitive appraisal in emotion. J. Pers. Soc. Psychol. 48, 813–838. doi: 10.1037/0022-3514.48.4.813 3886875

[ref68] Sornsenee P. Kongtragulsub K. Watcharajiranich K. Chantanuwat R. Aungchayakul A. Mangkhalathat K.. (2022). Factors associated with anxiety and depression among micro, small, and medium enterprise restaurant entrepreneurs due to Thailand? S COVID-19-related restrictions: a cross-sectional study. Risk Manag. Healthc. P. 5, 1157–1165. doi: 10.2147/RMHP.S359507, PMID: 35655746 PMC9153940

[ref69] Spielberger C. D. Anton W. D. Bedell J. (1976). “Emotions and anxiety: new concepts, methods, and applications,” in The nature and treatment of test anxiety. eds. Zuckerman M. Spielberger C. D., (New Jersey: The Halsted Press) 317–345.

[ref70] Stephan U. (2018). Entrepreneurs’ mental health and well-being: a review and research agenda. Acad. Manage. Perspect. 32, 290–322. doi: 10.5465/amp.2017.0001

[ref71] Thompson N. A. van Gelderen M. Keppler L. (2020). No need to worry? Anxiety and coping in the entrepreneurship process. Front. Psychol. 11:398. doi: 10.3389/fpsyg.2020.00398, PMID: 32226405 PMC7080856

[ref72] Tomaka J. Blascovich J. Kibler J. Ernst J. M. (1997). Cognitive and physiological antecedents of threat and challenge appraisal. J. Pers. Soc. Psychol. 73, 63–72. doi: 10.1037/0022-3514.73.1.63, PMID: 9216079

[ref73] Torres O. Benzari A. Fisch C. Mukerjee J. Swalhi A. Thurik R. (2021). Risk of burnout in French entrepreneurs during the COVID-19 crisis. Small Bus. Econ. 58, 717–739. doi: 10.1007/s11187-021-00516-2 PMC8192221 38624594

[ref74] Turner J. Akinremi T. (2020). The business effects of pandemics – A rapid literature review. Available at: https://persistent-url (Accessed 16 April 2020).

[ref75] Vartanova O. Kolomytseva O. Bilyk V. Budnikevich I. Vasylchenko L. Burtseva T. (2021). Enterprise competitive positioning based on knowledge resources identification. Entrep. Sustain. Iss. 9, 529–541. doi: 10.9770/jesi.2021.9.1(33)

[ref76] Wang S. Y. Liu Y. Du Y. Y. Wang X. Y. (2021). Effect of the COVID-19 pandemic on consumers' impulse buying: the moderating role of moderate thinking. Int. J. Env. Res. Pub. He. 18:11116. doi: 10.3390/ijerph182111116, PMID: 34769636 PMC8583521

[ref77] Watson L. Spence M. T. (2007). Causes and consequences of emotions on consumer behaviour - a review and integrative cognitive appraisal theory. Eur. J. Marking. 41, 487–511. doi: 10.1108/03090560710737570

[ref78] Xia L. H. Luo B. A. Sun Y. (2020). How can entrepreneurs achieve success in chaos?: the effects of entrepreneurs’ effectuation on new venture performance in China. Kybernetes 49, 1407–1428. doi: 10.1108/K-01-2019-0035

[ref79] Xu X. Y. Huang D. Chen Q. R. (2021). Stress and coping among micro-entrepreneurs of peer-to-peer accommodation. Int. J. Hosp. Manag. 97:103009. doi: 10.1016/j.ijhm.2021.103009

[ref80] Xu Z. D. Jia H. Q. (2022). The influence of COVID-19 on entrepreneur's psychological well-being. Front. Psychol. 12:823542. doi: 10.3389/fpsyg.2021.823542, PMID: 35095701 PMC8795827

[ref81] Yin J. Ni Y. (2021). COVID-19 event strength, psychological safety, and avoidance coping behaviors for employees in the tourism industry. J. Hosp. Tour. Manag. 47, 431–442. doi: 10.1016/j.jhtm.2021.04.017

[ref82] Zhang D. J. Fang Y. K. (2022). Are environmentally friendly firms more vulnerable during the COVID-19 pandemic? J. Clean. Prod. 355:131781. doi: 10.1016/j.jclepro.2022.131781, PMID: 35466288 PMC9015965

[ref83] Zhang W. Xiong S. K. Zheng Y. L. H. Wu J. N. (2022). Response efficacy and self-efficacy mediated the relationship between perceived threat and psychic anxiety among college students in the early stage of the COVID-19 pandemic. Int. J. Env. Res. Pub. He. 19:2832. doi: 10.3390/ijerph19052832, PMID: 35270528 PMC8910033

[ref84] Zutshi A. Mendy J. Sharma G. D. Thomas A. Sarker T. (2021). From challenges to creativity: enhancing SMEs’ resilience in the context of COVID-19. Sustainability-basel. 13:6542. doi: 10.3390/su13126542

